# Incidence and Factors Associated with Falls in Older People in a Long-Term Care Facility: A Prospective Study in Taiwan

**DOI:** 10.3390/healthcare12100959

**Published:** 2024-05-07

**Authors:** Hung-Chun Lee, Chia-Jung Hsieh, Jih-Shuin Jerng

**Affiliations:** 1 Department of Nursing, National Taiwan University Hospital, Taipei 100225, Taiwan; 2 School of Nursing, College of Nursing, National Taipei University of Nursing and Health Sciences, Taipei 112303, Taiwan; 3 Department of Internal Medicine, National Taiwan University Hospital, Taipei 100225, Taiwan; 4 Center for Quality Management, National Taiwan University Hospital, Taipei 100225, Taiwan

**Keywords:** institutional resident, fall, risk factor

## Abstract

Background: The effectiveness of applying a fall-risk assessment to prevent falls in residents of long-term care facilities has not been investigated. Methods: This prospective study enrolled elderly residents in a long-term care facility in Taiwan. Caregivers were provided with a health-status assessment and fall-risk data to enhance their fall-prevention practices. A multivariate analysis was performed to identify the factors associated with falls. Results: A total of 123 subjects, including 68 and 55 for general and nursing-care models, respectively, were assessed. Their health status and risk of falls were provided to the care units to enhance their fall-prevention practices. Subjects in the nursing-care model had more dementia and more prescribed medications, worse physiologic conditions, and higher fall risk. Of them, 28 (23%) had subsequent falls. A univariate analysis showed that those with and without falls were similar in demographic characteristics, prescribed medications, physiologic function, and fall risk. There was a tendency for more falls in the nursing-care model residents (accounting for 61% of those who fell; *p* = 0.053). A regression analysis showed that gender (beta = 1.359; 95% confidence interval = 0.345–2.374; *p* = 0.010) and NPI score (beta = 0.101; 95% CI = 0.001–0.200; *p* = 0.047) were associated with the risk of falls. Conclusion: Residents at the long-term care facility had a significant risk of falls despite knowledge of their fall risk and the implementation of preventive measures. In this context of being aware of the risk, gender, and psychiatric symptoms were significantly associated with falls. Caregivers at long-term care facilities should implement further measures to prevent falls based on behavioral and psychological symptoms.

## 1. Introduction

The prevention of falls is a top priority in caring for older people, and this concern becomes increasingly crucial as the global population of older people grows [1]. The incidence of falls among older individuals residing in long-term care facilities has been reported to be three times higher than that among community-dwelling seniors, significantly impacting their quality of life [2]. Another study revealed that one in five residents in long-term care facilities has experienced at least one fall, with an occurrence rate ranging from 10% to 25%, emphasizing the importance of post-fall assessment [3]. Residents in long-term care facilities are typically older people and frail, often having poor balance and an unsteady gait, leading to a higher risk of fall-related injuries. There is a risk of falling in 40% to 60% of patients with cardiovascular disease [4]. Implementing targeted interventions for older adults in different regions has proven more effective in reducing the risk of falls [5]. Chronic illnesses, which are particularly associated with older age, are linked to a higher incidence of falls [6]. Conditions such as Parkinson’s disease or dementia are also contributing factors, where the use of Alzheimer’s medications has been associated with a reduced risk of falling [7]. Medications for cerebral circulation and diuretics have been identified as potential contributors to an increased fall risk [8,9].

In addition to physical factors, residents in long-term care facilities often face cognitive impairment, negative emotions, memory issues, sleep disturbances, and restlessness, all of which impact daily life activities. Cognitive stimulation therapy (CST) on dementia patients has shown improvements in cognitive ability, quality of life, language, and activities of daily living [10]. These challenges cause distress and harm not only to the affected individuals but also to their families, other residents, and caregivers. Advanced age, lower education, hypertension, Parkinsonism, respiratory disease, stroke, and intractable epilepsy are associated with an increased risk of dementia [11,12]. Falls and fall-related injuries are the primary health concerns for residents of care facilities, often resulting in fractures. This issue poses the most crucial challenge in geriatric care, and multifactorial fall-prevention programs have proven effective in reducing the incidence of falls [13,14]. The residents of care facilities have more serious fall-related injuries than community-dwelling older people [12]. In addition, impaired mobility leads to a higher level of dependence and a lower level of self-confidence, which can affect follow-up care, increase care costs, and lead to social withdrawal and a decreased quality of life.

A recent systematic review and meta-analysis article pointed out that 18 prospective studies of older adults living in nursing homes found that risk factors were strongly associated with all falls, including past fall history and impaired ADL performance, insomnia, and depression.

Furthermore, the more older individuals are impaired in daily life, the more afraid they will be of falling. This indicates a direct correlation between physical health and mental health in older individuals who fall. The self-assessed needs of older adults with regard to physical, psychological, and social care have been reported to be higher one month after a fall compared to when they are discharged from the hospital after the fall [13]. Therefore, physical care after a fall and the prevention of additional falls can increase the physical aspects of the quality of life. In addition, family and society play an essential role in the satisfaction of care of older people. Encouragement from family and friends and the use of walking aids after an injury can help increase satisfaction in the quality of life of older individuals and reduce fear, and this, in turn, will lower health expenditure and human resources [2].

The prevention of falls and fall-related injuries in patients in different healthcare settings has been studied. In Taiwan, long-term care facilities are mostly in the private sector, with human resources for care providers adjusted based on the care need, such as nursing homes, care centers, and dementia care units. However, the function of residents admitted to these care facilities often decreases as their length of stay increases, resulting in an increased fall risk and fall events. Previous studies in Western countries have reported that falls at these facilities are associated with gender, comorbidities, physiological function, psychological status, and cognitive function [7]. However, reports on the factors associated with falls in long-term care facilities in Asia are lacking. Therefore, the aim of this study was to explore the factors associated with falls in residents living in a long-term care facility in Taiwan.

## 2. Methods

### 2.1. Study Design and Setting

This prospective study was conducted in two phases. The first phase involved the collection of cross-section data and analysis of the admitted residents, and the second phase involved analysis after one year of follow-up. The Research Ethics Committee B of National Taiwan University Hospital, Taipei, Taiwan approved this study (#201404033RINB on 10 June 2014). Written informed consent was obtained from the participants or their surrogates.

### 2.2. Participants

Residents of a long-term care facility in Northern Taiwan were screened using purposive sampling for eligibility for this study. The residents were divided into general-care and nursing-care units according to their condition. Residents who fulfilled all of the following criteria were recruited: (1) age older than 65 years; (2) had lived in the institution for at least one month; (3) a Mini-Mental State Examination (MMSE) score ≥ 10 [15]; (4) ability to verbalize and communicate; and (5) if a subject had cognitive impairment (Alzheimer’s or vascular dementia), a diagnosis from a physician was required. Further, subjects were included if they met the diagnostic criteria of the American Psychiatric Association (DSM-IV) and had a CDR of 1–2 [16]. The exclusion criteria were: (1) those who were unconscious; (2) those with vision or hearing impairment; (3) those with neuromusculoskeletal disorders; (4) those who had experienced emergency hospitalization in the past six months; and (5) those who could not be evaluated for balance or gait.

Health status and fall-risk assessment data were provided to the clinical caregivers, who then implemented necessary fall-prevention measures, including increased patient visits (nighttime and early morning), modification of the bathing environment, adequate restraints on chairs, use of walking aids, physical training, and repeated education for the residents.

Based on a dimensional analysis [17], a linear multiple regression analysis, which focuses on correlation and regression analysis of data, was used. Based on previous research experience, the sample size of this study was calculated using G-Power 3.1 statistical software. The setting conditions were F test: linear multiple regression, medium effect size = 0.15, α error = 0.05, power = 0.8, and predictor = 9. The sample size was at least 114 people.

### 2.3. Data Collection

Data collection focused on different groups of residents receiving nursing care, treatment, or dementia care and explored the factors associated with the risk of falls. The number of chronically ill patients at the facility was recorded. The investigators visited every resident, performed interviews and assessments, and collected the following data. (1) Basic characteristics, including age, gender, type of care received, whether the subject had dementia or Parkinson’s disease, and whether or not the subject was taking cerebral circulation drugs or diuretics were collected. (2) Fall-risk score was calculated using the Hendrich II Fall Risk Model, in which the total score of this assessment scale is 8 points. The higher the score, the higher the risk of falling [18]. (3) Health condition was assessed using the Barthel Activities of Daily Living (ADL) index [19]. The interpretation of the ADL index score is as follows. The degree of dependence on the Barthel index was divided into six categories: 1. fully dependent (0~20), 2. severe dependence (21~40), 3. moderately dependent (41~60), 4. mild dependent (61~80), 5. independent (81~90), and 6. fully independent (91~100). The MMSE Chinese version has a maximum score of 30 points [20] for the Geriatric Depression Scale-Short Form (GDS) Chinese version, and the range of scores on the GDS was from 0–15 [21]. For the Cornell Scale for Depression in Dementia (CSDD), the scale consists of 19 items. A higher total scale score indicates more severe depressive symptoms [22]. For the Neuropsychiatric Inventory (NPI) Chinese version, it assesses 12 different neuropsychiatric disturbances, and the range of scores on NPI was from 0–36 [23]. (4) Co-morbidities and (5) medications information was also collected.

### 2.4. Statistical Analysis

We first performed a descriptive analysis of the characteristics of the included subjects, and then a multiple regression to analyze the factors associated with the risk of falls. SPSS 20.0 software (SPSS Inc., Chicago, IL, USA) was used to conduct the statistical analysis, including descriptive statistics, independent-samples t-test, analysis of variance, Pearson correlation coefficients, and multivariate logistic regression. A *p*-value of less than 0.05 was statistically significant. We adopted the univariate comparisons of demographic data, physiologic assessments, and fall-risk scores between those with and without fall events in the nursing-care model, as well as the multivariate regression analysis for the factors associated with fall events. This study employs the variance inflation factor (VIF) to determine whether there is a collinearity issue. When the VIF value is less than 10, it indicates that there is no significant collinearity problem.

## 3. Results

During the study period, a total of 123 subjects, including 68 residents for the general-care model and 55 for the nursing-care model, were recruited. Their individual data on physiologic function and fall-risk assessments were passed to their caregiver, who then implemented fall-prevention measures. Table 1 summarizes these features and comparisons between the subjects in the general- and nursing-care models. The results showed that the subjects in the nursing-care model had a higher rate of dementia (36.4% vs. 1.5%, *p* < 0.001), lower rate of osteoporosis (5.5 vs. 20.6%, *p* = 0.016), fewer spine disorders (0% vs. 10.3%, *p* = 0.014), lower balance function (11.11 ± 4.1 vs. 12.81 ± 3.28, *p* = 0.014), lower cognitive function (25.47 ± 4.89 vs. 28.07 ± 2.67, *p* = 0.001), and a higher fall-risk score (1.73 ± 1.27 vs. 1.24 ± 1.01, *p* = 0.021) than the general-care model residents.

Of the 123 subjects, 28 (22.8%) experienced at least one fall event during their stay at the facility, even though the caregivers had been provided with the subjects’ data regarding physiologic function and fall risk. Table 2 summarizes the univariate comparisons of demographic data, physiologic assessments, and fall-risk scores between those with and without fall events. There were no significant differences in demographic data, co-morbidities, prescribed medications, physiologic assessment data, and fall-risk scores between those with and without falls. There was a tendency toward more falls in the nursing-care model residents (30.9% vs. 16.2%, *p* = 0.053).

There were no significant differences in demographic data, comorbidities, prescribed medications, physiologic function, and fall-risk scores between the two groups (Table 3).

We found that gender (beta = 1.359; 95% confidence interval = 0.345–2.374; *p* = 0.010) and NPI score (beta = 0.101; 95% CI = 0.001–0.200; *p* = 0.047) were associated with the risk of falls (*R*^2^ = 0.653, *p* < 0.001). Other functional and physiologic scores were not associated with falls in this cohort (Table 4).

## 4. Discussion

In this study, we found that the subjects at the long-term facility cared for under the general-care and nursing-care models still had a high incidence of falls (22.8%) even though the caregivers were provided with the results of a panel of physiologic and fall-risk assessments. Similar results found that the incidence of falls in older adults living in nursing homes is higher than in a prior meta-analysis of prospective study designs [13]. Therefore, older residents have a variety of risk factors for falls, so we need to provide a protective living environment. We also found that the subjects with and without subsequent fall events were similar with regard to their demographic features, comorbidities, prescribed medications, physiologic function, and fall-risk score. A regression analysis showed that only gender and NPI score were associated with falls, whereas the fall-risk score was not a significant factor. Although our research findings showed the main risk of falls factors among residents at the long-term care facility compared with the previous meta-study analyses [2,13], some of the variables we found are not significant. There is an important reason, which may be that the residents’ health outcomes and functional status interfere with each other, so the prevention strategies for long-term care institutional residents should also be considered holistically in order to minimize the adverse consequences of falls for older adults.

The demographic characteristics in this study were similar to reports in the literature [23,24], in that the subjects were older. However, the factors associated with falls previously reported in the literature, including age [7,15], cognitive impairment [15], bodily functions [25], medications [8,9], and chronic diseases [6,7], were not significant in the present study. On the other hand, psychiatric symptoms [7] were associated with falls, as shown by the NPI score. As these factors also contributed to a higher Hendrich fall-risk score, caregivers can implement preventive care processes to reduce falls based on the information from these assessments, as noted in this study. Walking, lower-limb muscle toning, and balance training have been reported to reduce the risk of falls [26]. These conditions could be managed adequately by care providers. Our study cohort was older residents in a long-term facility who reported insignificant factors for falls, including level of education [6], length of residency [27], depression [2], and past fall experience [24,27].

Fall prevention may need to include interventions to improve physical function. Exercise regimes have been reported to be suitable for such residents, such as lower-limb muscle toning and balance training [28] and balance and gait training [24,29]. On the other hand, non-modifiable factors, such as gender, and difficult-to-modify factors, such as psychiatric symptoms, as suggested in the literature [6,7,23], may remain a challenge. Lee et al. [30] reported that male residents with cognitive impairment had a relatively higher risk of falls than females. This is consistent with the results of the present study. Based on our findings, we suggest, that to reduce the risk of falls, stress should be put on care education, exercises that train lower-limb muscles, devices such as handrails and crutches, sensitivity to gender and minor details, and correctly evaluating cognitive function and psychiatric symptoms. A complete evaluation of all areas can help detect risks early and determine the likelihood of falls. Managers of care facilities should regularly follow up and analyze the incidence of falls of different types in their residents, particularly those with multiple falls. Fall-prevention seminars may also be beneficial for caregivers. Care facilities may need to actively screen for fall risks in older adults, followed by comprehensive evaluations and interventions.

There are several limitations to this study. First, this was a single-center cross-sectional study with a relatively limited number of cases, so that the number of falls may be relatively small, and the assessment of fall-risk factors may have low power. We suggest that fall-risk follow-up and management should be implemented in care facilities around the country, which will help develop clinical guidelines that can prevent falls for older residents in care facilities and provide interventions when necessary. Second, the interventions to prevent falls in the subjects were partly affected by the investigators of this study, as the results of health condition and fall-risk scores were provided to the caregivers and administrative staff of the facility. Therefore, it is recommended that future research designs include a control group to collect cases. Third, the length of stay in the long-term facility may affect the fall risk. Therefore, our single assessment results may not be able to predict the long-term outcomes if the health conditions change. Periodic reassessments of the fall risk may be needed.

## 5. Conclusions

In conclusion, the residents at the long-term care facility with different health conditions had a significant risk of falls, even though the care providers knew the risks and took preventive measures. In this context of being aware of the risk, gender and psychiatric symptoms were significant factors associated with falls. Caregivers at long-term care facilities may implement further measures to prevent falls based on behavioral and psychological symptoms.

## Figures and Tables

**Table 1:** Demographic features, health condition, and risk of falls.

Variable	Total (*n* = 123)	General Care (*n* = 68)	Nursing Care (*n* = 55)	χ^2^/*t*	Sig
Age (years)	85.33 ± 6.34	85.56 ± 6.15	85.05 ± 6.62	*t* = 0.265	0.663
Gender, male	34 (27.6%)	20 (29.4%)	14 (25.5%)	χ^2^ = 0.238	0.626
Co-morbidities					
Hypertension	101 (82.1%)	58 (85.3%)	43 (78.2%)	χ^2^ = 1.047	0.306
Other cardiovascular diseases	49 (39.8%)	28 (41.2%)	21 (38.2%)	χ^2^ = 0.114	0.736
Diabetes mellitus	37 (30.1%)	18 (26.5%)	19 (34.5%)	χ^2^ = 0.943	0.332
Dementia	21 (17.1%)	1 (1.5%)	20 (36.4%)	χ^2^ = 26.148	<0.001
Impaired vision	18 (14.6%)	13 (19.1%)	5 (9.1%)	χ^2^ = 2.447	0.118
Osteoporosis	17 (13.8%)	14 (20.6%)	3 (5.5%)	χ^2^ = 5.847	0.016
Psychiatric diseases	8 (6.5%)	5 (7.4%)	3 (5.5%)	χ^2^ = 0.180	0.671
Stroke	7 (5.7%)	3 (4.4%)	4 (7.3%)	χ^2^ = 0.464	0.496
Spine disorders	7 (5.7%)	7 (10.3%)	0 (0%)	χ^2^ = 6.003	0.014
Parkinson’s disease	5 (4.1%)	1 (1.5%)	4 (7.3%)	χ^2^ = 2.625	0.105
Total hip-replacement history	5 (4.1%)	1 (1.5%)	4 (7.3%)	χ^2^ = 2.625	0.105
Total knee-replacement history	4 (3.3%)	3 (4.4%)	1 (1.8%)	χ^2^ = 0.650	0.420
Rheumatoid arthritis	3 (2.4%)	3 (4.4%)	0 (0%)	χ^2^ = 2.487	0.115
Malignancy	3 (2.4%)	3 (4.4%)	0 (0%)	χ^2^ = 2.487	0.115
Prescribed medications (*n* = 35)	35 (28.5%)	7 (10.3%)	28 (50.9%)	χ^2^ = 5.925	<0.001
Antihypertensives	27 (22.0%)	7 (10.3%)	20 (36.4%)	χ^2^ = 2.593	0.107
Sedatives/hypnotics	24 (19.5%)	5 (7.4%)	19 (34.5%)	χ^2^ = 0.033	0.856
Other cardiovascular medications	14 (11.4%)	3 (4.4%)	11 (20.0%)	χ^2^ = 0.030	0.863
Laxatives	11 (8.9%)	3 (4.4%)	8 (14.5%)	χ^2^ = 0.530	0.466
Anti-diabetic drugs	10 (8.1%)	1 (1.5%)	9 (16.4%)	χ^2^ = 0.875	0.350
Cerebral vascular drugs	8 (6.5%)	0 (0%)	8 (14.5%)	χ^2^ = 2.593	0.107
Vitamins	7 (5.7%)	0 (0%)	7 (12.7%)	χ^2^ = 2.188	0.139
Diuretics	6 (4.9%)	1 (1.5%)	5 (9.1%)	χ^2^ = 0.050	0.823
Anti-depression agents	4 (3.3%)	1 (1.5%)	3 (5.5%)	χ^2^ = 0.071	0.791
Anti-epileptic agents	3 (2.4%)	0 (0%)	3 (5.5%)	χ^2^ = 0.820	0.365
Health and fall-risk scores					
Physical function (Barthel)	5.13 ± 0.88	5.25 ± 0.74	4.98 ± 1.01	*t* = 0.440	0.092
Balance function (Tinetti)	12.05 ± 3.75	12.81 ± 3.28	11.11 ± 4.1	*t* = 4.103	0.014
Gait (Tinetti)	9.96 ± 2.07	10.18 ± 1.83	9.69 ± 2.32	*t* = 1.314	0.196
Depression (GDS)	11.69 ± 1.3	11.64 ± 1.1	11.81 ± 1.68	*t* = 9.172	0.621
Depression (Cornel)	4.36 ± 5.57	5.00 ± 0.00	4.33 ± 5.69	*t = 0.115*	0.910
Cognitive function (MMSE)	26.91 ± 4.03	28.07 ± 2.67	25.47 ± 4.89	*t* = 21.031	<0.001
Behavioral and psychological symptoms (NPI)	9.88 ± 4.40	8.00 ± 0.00	9.96 ± 4.48	*t* = −0.429	0.672
Hendrich fall-related risk score	1.46 ± 1.15	1.24 ± 1.01	1.73 ± 1.27	*t* = 5.008	0.021
Occurrence of fall events	28 (22.8%)	11 (16.8%)	17 (30.9%)	χ^2^ = 3.754	0.053

**Table 2:** Comparisons between the residents with and without fall events.

Variable	Total (*n* = 123)	Fall (−) (*n* = 95)	Fall (+) (*n* = 28)	χ^2^/*t*	Sig
Age (years)	85.33 ± 6.34	85.09 ± 6.02	86.14 ± 7.38	*t* = 1.507	0.444
Gender, male	34 (27.6%)	29 (30.5%)	5 (17.9%)	χ^2^ = 1.735	0.188
Care setting				χ^2^ = 3.754	0.053
General care	68 (55.3%)	57 (60.0%)	11 (39.3%)		
Nursing care	55 (44.7%)	38 (40.0%)	17 (60.7%)		
Co-morbidities					
Hypertension	101 (82.1%)	79 (83.2%)	22 (78.6%)	χ^2^ = 0.310	0.578
Other cardiovascular diseases	49 (39.8%)	41 (43.2%)	8 (28.6%)	χ^2^ = 1.920	0.166
Diabetes mellitus	37 (30.1%)	26 (27.4%)	11 (39.3%)	χ^2^ = 1.460	0.227
Dementia	21 (17.1%)	15 (15.8%)	6 (21.4%)	χ^2^ = 0.486	0.486
Impaired vision	18 (14.6%)	15 (15.8%)	3 (10.7%)	χ^2^ = 0.446	0.504
Osteoporosis	17 (13.8%)	12 (12.6%)	5 (17.9%)	χ^2^ = 0.496	0.481
Psychiatric diseases	8 (6.5%)	5 (5.3%)	3 (10.7%)	χ^2^ = 1.057	0.304
Stroke	7 (5.7%)	4 (4.2%)	3 (10.7%)	χ^2^ = 1.704	0.192
Spine disorders	7 (5.7%)	5 (5.3%)	2 (7.1%)	χ^2^ = 0.142	0.706
Parkinson’s disease	5 (4.1%)	3 (3.2%)	2 (7.1%)	χ^2^ = 0.881	0.348
Total hip-replacement history	5 (4.1%)	4 (4.2%)	1 (3.6%)	χ^2^ = 0.023	0.880
Total knee-replacement history	4 (3.3%)	4 (4.2%)	0 (0%)	χ^2^ = 1.219	0.270
Rheumatoid arthritis	3 (2.4%)	2 (2.1%)	1 (3.6%)	χ^2^ = 0.195	0.658
Malignancy	3 (2.4%)	1 (1.1%)	2 (7.1%)	χ^2^ = 3.371	0.066
Prescribed medications	35 (28.5%)	27 (28.4%)	8 (28.6%)		
Antihypertensives	27 (22.0%)	21 (22.1%)	6 (21.4%)	χ^2^ = 0.027	0.869
Sedatives/hypnotics	24 (19.5%)	20 (21.0%)	4 (14.3%)	χ^2^ = 1.660	0.198
Other cardiovascular medications	14 (11.4%)	11 (11.6%)	3 (10.7%)	χ^2^ = 0.027	0.869
Laxatives	11 (8.9%)	8 (8.4%)	3 (10.7%)	χ^2^ = 0.177	0.674
Anti-diabetic drug	10 (8.1%)	8 (8.4%)	2 (7.1%)	χ^2^ = 0.065	0.799
Cerebral vascular drugs	8 (6.5%)	6 (6.3%)	2 (7.1%)	χ^2^ = 0.027	0.869
Vitamins	7 (5.7%)	6 (6.3%)	1 (3.6%)	χ^2^ = 0.365	0.546
Diuretics	6 (4.9%)	3 (3.2%)	3 (10.7%)	χ^2^ = 3.026	0.082
Anti-depression agents	4 (3.3%)	3 (3.2%)	1 (3.6%)	χ^2^ = 0.012	0.914
Anti-epileptic agents	3 (2.4%)	3 (3.2%)	0 (0%)	χ^2^ = 0.972	0.324
Health and fall-risk scores					
Physical function (Barthel)	5.13 ± 0.88	5.14 ± 0.91	5.11 ± 0.79	*t* = 0.104	0.876
Balance function (Tinetti)	12.05 ± 3.75	11.98 ± 3.83	12.29 ± 3.56	*t* = 0.127	0.706
Gait (Tinetti)	9.96 ± 2.07	10.05 ± 1.94	9.64 ± 2.45	*t* = 0.922	0.422
Depression (GDS)	11.69 ± 1.3	11.75 ± 1.22	11.5 ± 1.57	*t* = 1.055	0.431
Depression (Cornel)	4.36 ± 5.57	4.84 ± 6.27	2.83 ± 1.94	*t* = 1.068	0.453
Cognitive function (MMSE)	26.91 ± 4.03	26.75 ± 4.15	27.46 ± 3.57	*t* = 0.202	0.410
Behavioral and psychological symptoms (NPI)	9.88 ± 4.40	9.74 ± 4.54	10.33 ± 4.27	*t* = 0.090	0.779
Hendrich fall-related risk score	1.46 ± 1.15	1.45 ± 1.23	1.46 ± 0.88	*t =* −0.047	0.0963

**Table 3:** Comparisons between the nursing-care unit residents with and without fall events.

Variable	Total (*n* = 55)	Fall (−) (*n* = 38)	Fall (+) (*n* = 17)	χ^2^/*t*	Sig
Age (years)	85.05 ± 6.62	84.63 ± 6.31	86 ± 7.37	*t* = 0.124	0.484
Gender, male	14 (25.5%)	11 (28.9%)	3 (17.6%)	χ^2^ = 0.790	0.374
Co-morbidities					
Hypertension	43 (78.2%)	30 (78.9%)	13 (76.5%)	χ^2^ = 0.042	0.837
Other cardiovascular diseases	21 (38.2%)	16 (42.1%)	5 (29.4%)	χ^2^ = 0.802	0.371
Diabetes mellitus	19 (34.5%)	12 (31.6%)	7 (41.2%)	χ^2^ = 0.478	0.489
Dementia	20 (36.4%)	14 (36.8%)	6 (35.3%)	χ^2^ = 0.012	0.912
Impaired vision	5 (9.1%)	3 (7.9%)	2 (11.8%)	χ^2^ = 0.213	0.645
Osteoporosis	3 (5.5%)	1 (2.6%)	2 (11.8%)	χ^2^ = 1.900	0.168
Psychiatric diseases	3 (5.5%)	3 (7.9%)	0 (0%)	χ^2^ = 1.420	0.233
Stroke	4 (7.3%)	2 (5.3%)	2 (11.8%)	χ^2^ = 0.736	0.391
Parkinson’s disease	4 (7.3%)	2 (5.3%)	2 (11.8%)	χ^2^ = 0.736	0.391
Total hip-replacement history	4 (7.3%)	4 (10.5%)	0 (0%)	χ^2^ = 1.930	0.165
Total knee-replacement history	1 (1.8%)	1 (2.6%)	0 (0%)	χ^2^ = 0.456	0.500
Prescribed medications	28 (50.9%)	20 (52.6%)	8 (47.1%)		
Antihypertensives	20 (36.4%)	14 (36.8%)	6 (35.3%)	χ^2^ = 0.070	0.791
Sedatives/hypnotics	19 (34.5%)	15 (39.5%)	4 (23.5%)	χ^2^ = 1.637	0.201
Other cardiovascular medications	11 (20.0%)	8 (21.0%)	3 (17.6%)	χ^2^ = 0.015	0.903
Laxatives	8 (14.5%)	5 (13.2%)	3 (17.6%)	χ^2^ = 0.438	0.508
Anti-diabetic drugs	9 (16.4%)	7 (18.4%)	2 (11.8%)	χ^2^ = 0.262	0.609
Cerebral vascular drugs	8 (14.5%)	6 (15.8%)	2 (11.8%)	χ^2^ = 0.070	0.791
Vitamins	7 (12.7%)	6 (15.8%)	1 (5.9%)	χ^2^ = 0.933	0.334
Diuretics	5 (9.1%)	2 (5.3%)	3 (17.6%)	χ^2^ = 2.946	0.086
Anti-depression agents	3 (5.5%)	2 (5.3%)	1 (5.9%)	χ^2^ = 0.037	0.847
Anti-epileptic agents	3 (5.5%)	3 (7.9%)	0 (0%)	χ^2^ = 1.337	0.246
Analgesics	2 (3.6%)	0 (0%)	2 (11.8%)	χ^2^ = 5.385	0.020
Health and fall-risk scores					
Physical function (Barthel)	4.98 ± 1.01	4.89 ± 1.06	5.18 ± 0.88	*t* = 0.009	0.343
Balance function (Tinetti)	11.11 ± 4.1	10.68 ± 4.26	12.06 ± 3.67	*t* = 0.699	0.255
Gait (Tinetti)	9.69 ± 2.32	9.74 ± 2.13	9.59 ± 2.76	*t* = 3.444	0.828
Depression (GDS)	11.81 ± 1.68	12 ± 1.41	11.45 ± 2.11	*t* = 2.394	0.397
Depression (Cornel)	4.33 ± 5.69	4.83 ± 6.46	2.83 ± 1.94	*t* = 1.226	0.468
Cognitive function (MMSE)	25.47 ± 4.89	25 ± 5.18	26.53 ± 4.14	*t* = 0.531	0.288
Behavioral and psychological symptoms (NPI)	9.96 ± 4.48	9.83 ± 4.66	10.33 ± 4.27	*t* = 0.050	0.819
Hendrich fall-related risk score	1.73 ± 1.27	1.76 ± 1.36	1.65 ± 1.06	*t* = 0.311	0.757

**Table 4:** Regression analysis for the risk of falls.

Variable	Beta	95% Confidence Interval of Beta	*t*	Sig	VIF
Gender	1.359	0.345–2.374	2.744	0.010	1.985
MMSE score	−0.067	−0.154–0.019	−1.594	0.122	1.264
NPI score	0.101	0.001–0.200	2.075	0.047	1.690
Barthel score	−0.284	−0.890–0.323	−0.957	0.347	3.831
Balance function (Tinetti)	−0.064	−0.239–0.110	−0.755	0.457	3.756
Gait (Tinetti)	0.136	−0.239–0.511	0.742	0.464	4.867

Notes: AIC: 14.57089; BIC: 11.37937; F-value: 8.777 (*p*-value < 0.001).

## Data Availability

All of the data used in this study are available from the authors upon reasonable request.
